# The Effect of Chitosan Gel 15% in the Surgical Treatment of Stage III Periodontitis: A Case Report of Two Cases

**DOI:** 10.7759/cureus.58965

**Published:** 2024-04-24

**Authors:** Ahmad M Alnada, Wael H Almahdi, Mohamad A Fawaz

**Affiliations:** 1 Periodontology, Damascus University, Damascus, SYR; 2 Periodontics, Damascus University, Damascus, SYR; 3 Public Health, Central Michigan University, Mount Pleasant, USA

**Keywords:** probing depth, periodontal pockets, open flap debridement, chitosan, bleeding on probing (bop)

## Abstract

Periodontal diseases are widely spread, particularly in adults. Chitosan has non-toxicity and biocompatibility properties, as it has been studied in many studies in various surgical applications. This case report includes two female patients (aged 23 and 48) who were treated by the application of Chitosan gel 15% during open flap debridement in an aggregate of 26 periodontal pockets. Several clinical measurements were evaluated (probing depth, gingival recession, and bleeding on probing) for the treated periodontal pockets, between two periods, the first in baseline and then after six months. The results showed a reduction in probing depth of (3.30±0.27) after six months. The bleeding on probing also decreased from 84.61% to 0%. This case report concluded that the application of Chitosan gel 15% reduced pocket depth and bleeding on probing when applied in open flap debridement.

## Introduction

Periodontal diseases are widespread around the world, and the incidence of periodontitis in adults reaches 35% worldwide [1]. Gingivitis is the primary form of periodontal disease. This infection is caused by plaque germs that accumulate on the gingival margin. Gingivitis does not affect the periodontal tissue and is completely reversible. However, periodontitis causes loss of attachment and absorption in the alveolar bone, which often causes tooth loss. The main feature of periodontal disease is loss of attachment, which clinically manifests as a periodontal pocket resulting from the separation of the gingiva from the tooth surface [2]. In addition to pathogenic organisms present in the plaque, genetic and environmental factors, especially smoking, play a contributing role in the occurrence of the disease. Dermatology syndromes and blood diseases can also have periodontal manifestations [3].

Treatment of periodontal tissue diseases involves many techniques and procedures and depends on the condition of the disease and the ultimate goal of treatment. Mechanical treatment is the cornerstone of periodontal treatment through removing plaque, scaling, and root planing when needed. As for drug treatment, it is a supportive treatment that does not replace mechanical treatment and may be used in a systemic or local form. Some moderate to severe cases are treated with a surgical procedure to reach the root surface and perform root debridement to reduce the depth of the pocket and return it to its natural state and allow for plaque to be removed [4].

Many topical agents are applied in conjunction with non-surgical and surgical treatment. These include topically applied antibiotics or agents that accelerate healing, such as platelet derivatives. The goal of this application is to reduce periodontal pathogenic organisms present in periodontal pockets or to reduce the consequences of surgical treatment such as gingival recession [5,6].

Chitin is the most widespread biopolymer in nature after cellulose and can be found in various species such as crustaceans, insects, and fungi [7]. Chitin is a polymer composed of N-acetyl-D-glucosamine, and when it undergoes deacetylation the repeating units in the polymer are mostly without the acetyl functional group (β-1, 4-D) glucosamine. The polymer formed is Chitosan [8].

## Case presentation

Chitosan gel 15% preparation

Chitosan gel is formulated by mixing 15g of Chitosan powder (Sigma Aldrich^®^, St. Louis, USA) with 85g of sterile water whose pH was adjusted to be below 7 using citric acid [9].

Case 1

**Figure 1:**
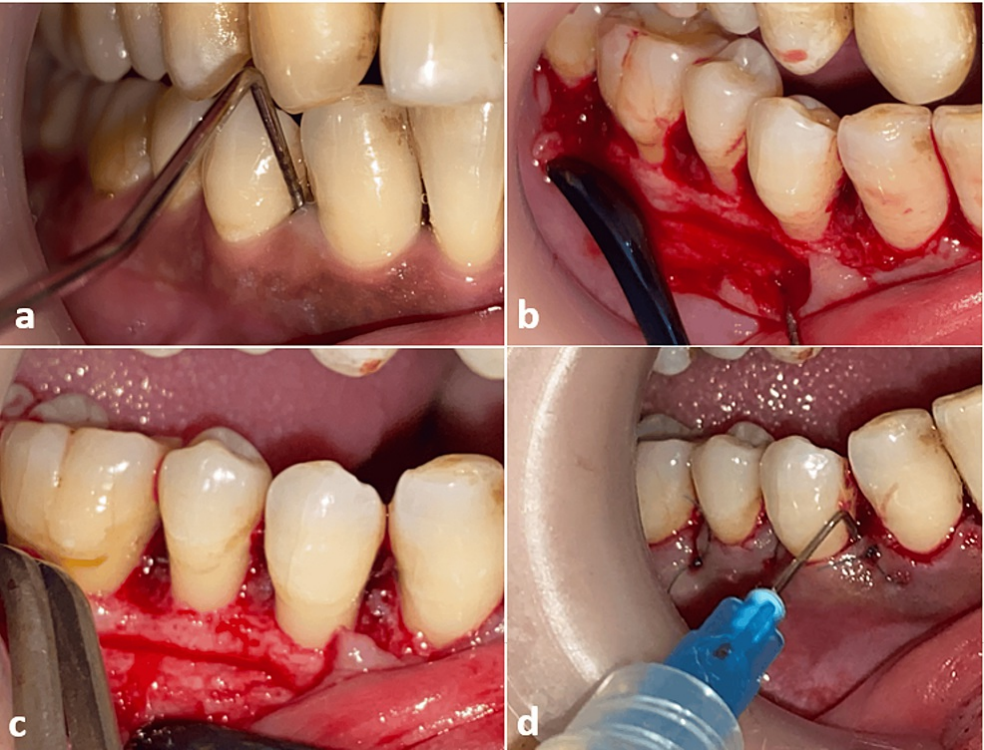
The surgical procedure (OFD) of case 1: (a) probing depth at baseline; (b) flap elevation; (c) roots after debridement; (d) Chitosan gel injection after flap sutured. OFD: open flap debridement

A 48-year-old female patient diagnosed with (Stage III - Grade A) periodontitis has 14 periodontal pockets larger than 5 mm in the mandible. She is a non-smoker and does not suffer from any systemic diseases or drug allergies. She was treated by scaling and root planning, and after four weeks, an open flap debridement was performed in the periodontal pockets (Figure 1), in which Chitosan gel 15% was applied within the pockets before and after suturing using 4-0 nylon threads. This process was performed on all periodontal pockets. The probing depth, bleeding on probing, and gingival recession were measured using a William probe (JK Surgical, Karachi, Pakistan). The patient was re-evaluated six months after surgery.

**Figure 2:**
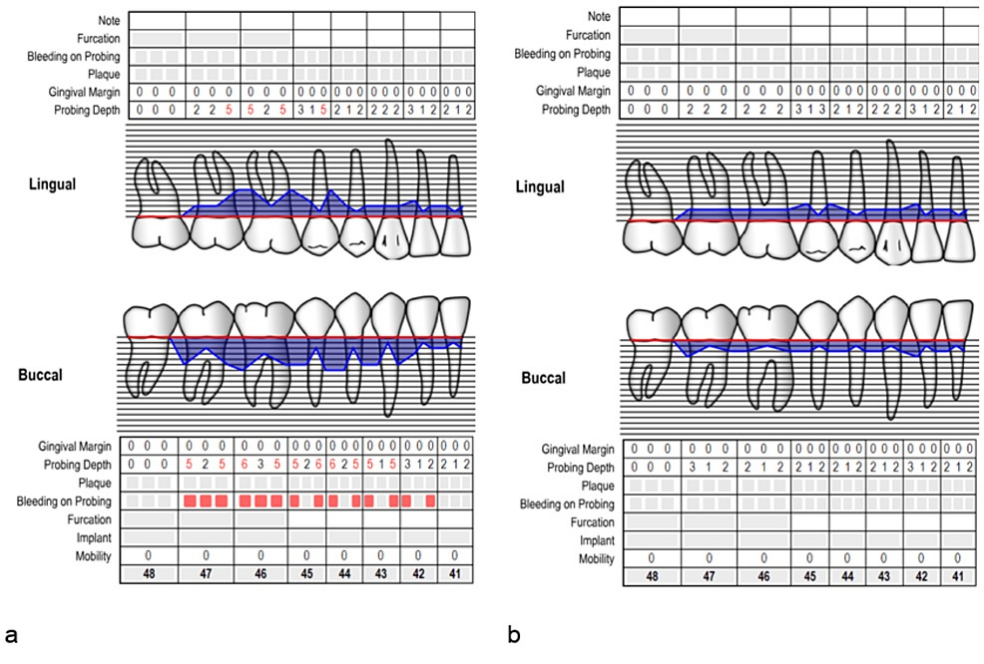
The periodontal chart for case 1: (a) at baseline, (b) after six months.

The periodontal measurements were recorded in the online periodontal chart (Figure 2).

Case 2

**Figure 3:**
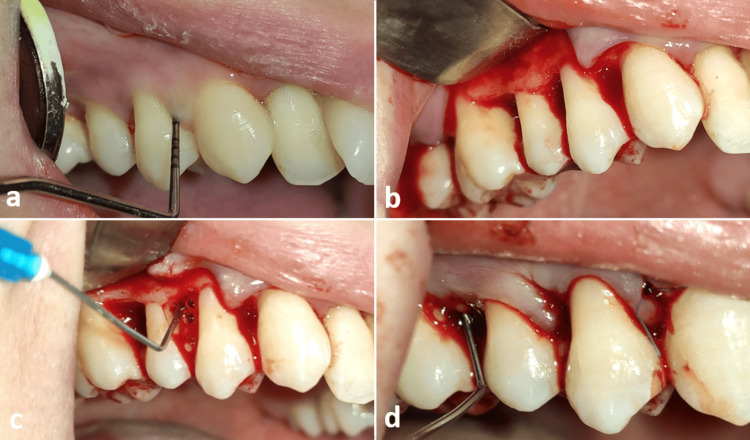
The surgical procedure (OFD) of case 2: (a) probing depth at baseline; (b) flap elevation; (c) Chitosan gel injection after debridement; (d) Chitosan gel injection after flap sutured. OFD: open flap debridement

A 23-year-old female patient diagnosed with (Stage III - Grade A) periodontitis has 12 periodontal pockets larger than 5 mm in the mandible. She is a non-smoker and does not suffer from any systemic diseases or drug allergies. She was treated by scaling and root planning. After four weeks, an open flap debridement was performed in the periodontal pockets (Figure 3) in which Chitosan gel 15% was applied within the pockets before and after suturing using 4-0 nylon threads. This was performed on all periodontal pockets. The probing depth, bleeding on probing, and gingival recession were measured using a William probe. The patient was re-evaluated six months after surgery.

**Figure 4:**
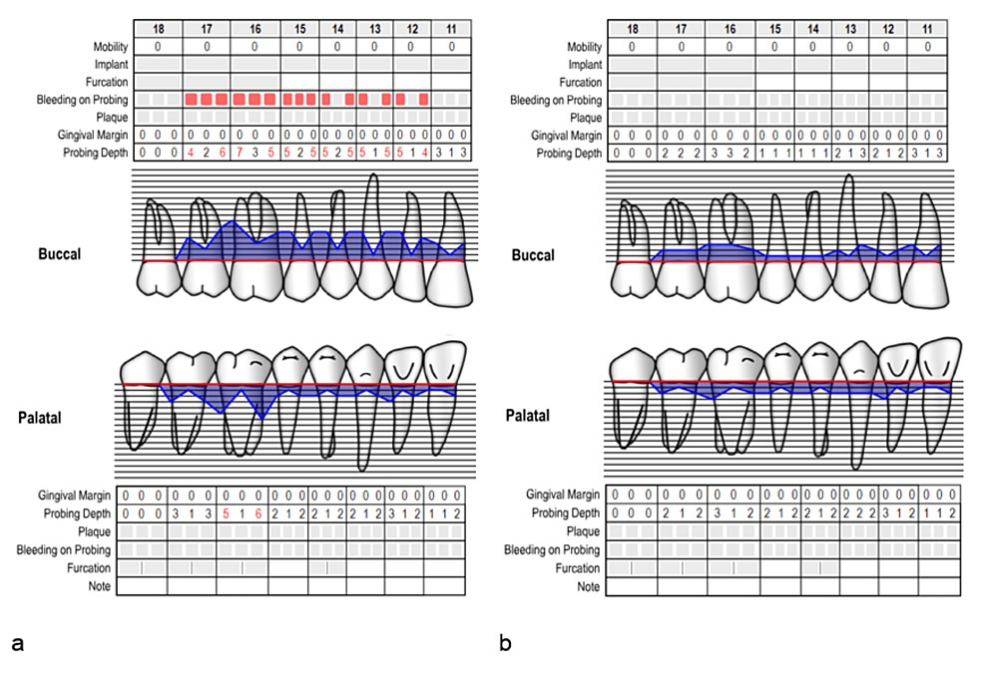
The periodontal chart for case 2: (a) at baseline, (b) after six months.

The periodontal measurements were recorded in the online periodontal chart (Figure 4).

Outcomes of the previous cases

This case report included two female patients aged 23 and 48 who were treated by the application of Chitosan gel 15% during open flap debridement in an aggregate of 26 periodontal pockets. Several clinical measurements were evaluated for the treated periodontal pockets between the two periods: the first in baseline and after six months. The results showed a reduction in probing depth, as its average in the two previous cases reached 5.26±0.53 mm at the baseline and became 1.96±0.59 mm after six months, with a reduction of 3.30±0.27 mm. The two previous cases showed a recession in only two periodontal sites of 1 mm for each recession of 26 sites. The average after six months was 0.07±0.27 mm. The bleeding on probing also decreased from 84.61% to 0%. Table 1 shows the descriptive statistics for the studied measurements.

**Table 1:** Mean of measured parameters in the above two cases

Measurements	Baseline	Six Months
Probing Depth (mm)	5.26±0.53	1.96±0.59
Gingival Recession (mm)	0.00	0.07±0.27
Percentage of Bleeding on Probing	84.61%	0%

## Discussion

Chitosan was chosen due to its non-toxicity and biocompatibility, as it has been studied in many studies in various surgical applications, such as its use as an absorbable barrier membrane [10], its use as a scaffold material for bone grafts [11], or its use as a gel that is injected during the non-surgical treatment of supra bony periodontal pockets [12].

Chitosan has antioxidant properties through its bonding to free radicals inside and outside cells and neutralizes the damage caused by these free radicals in the periodontal tissue which, in turn, contributes to attachment loss. It also enhances and accelerates healing by stimulating macrophages and B-cells to secrete antibodies [13]. The most important effect of Chitosan is its antimicrobial effect, especially in bacteria. It is important to eliminate periodontal pathogenic organisms. The effects of Chitosan have been tested in vitro on many periodontal pathogenic bacteria, and Chitosan showed an inhibition of them and an anti-bacterial effect on these organisms [14]. Many studies in the medical literature have shown the effect of Chitosan gel at different concentrations in reducing the depth of the periodontal pocket and increasing clinical attachment [9,12,15].

## Conclusions

This case report concluded that the application of Chitosan gel 15% enhances the clinical indices. The application of Chitosan gel 15% reduced pocket depth and reduced the active inflammation by reducing bleeding on probing when applied in open flap debridement.
